# Exogenous lipoid pneumonia masquerading as a pulmonary nodule: a case report

**DOI:** 10.3389/fmed.2025.1723734

**Published:** 2026-01-05

**Authors:** Yao Shen, Su Yao

**Affiliations:** Department of Pathology, Guangdong Provincial People's Hospital and Guangdong Academy of Medical Sciences, Southern Medical University, Guangzhou, China

**Keywords:** exogenous lipoid pneumonia, lung cancer, pathological morphology, misdiagnosis, pulmonary nodule

## Abstract

This case report describes a patient with exogenous lipoid pneumonia (ELP) presenting as a solitary pulmonary nodule. ELP is a rare chronic pulmonary disorder characterized by nonspecific clinical symptoms and imaging findings. When manifesting as a mass or nodule, features such as lobulation and spiculation on imaging can closely resemble lung cancer, frequently leading to misdiagnosis and unnecessary surgical intervention. We present a retrospective analysis of a pathologically confirmed case of ELP to improve recognition of this condition among both clinicians and pathologists.

## Case report

**Figure 1:**
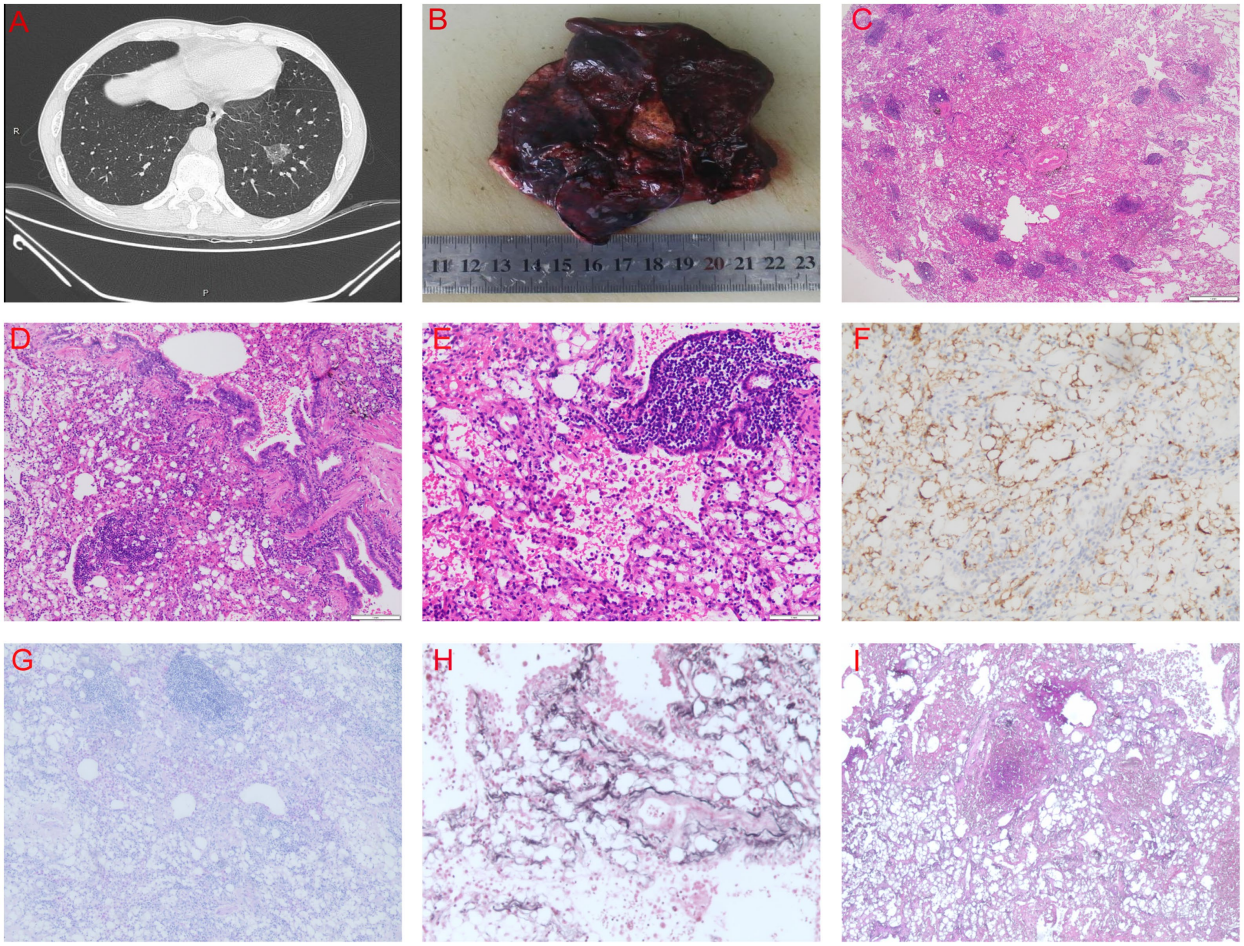
CT and pathology images. **(A)** Chest spiral CT scan. **(B)** Gross appearance of the left lower lung nodule. **(C–E)** Hematoxylin and eosin (H&E) staining showing extensive histiocytic hyperplasia with lipoid-laden vacuoles and lipoid deposits in the bronchial lumens, alveolar spaces, and lung interstitium. **(F)** Immunohistochemical staining for CD163, demonstrating abundant vacuolated histiocytic hyperplasia. **(G–I)** Special staining results: **(G)** PAS (+), **(H)** PM (−), **(I)** GS (−).

A 66-year-old man was referred to our hospital following the detection of a nodule in the left lower lung during a routine health examination 20 days earlier. Chest computed tomography (CT) revealed a 26-mm mixed ground-glass opacity nodule in the anteromedial basal segment of the left lower lobe. The nodule was well-demarcated but exhibited classic features of malignancy: a spiculated margin with radiating strands and a lobulated contour. Internally, solid components accounted for approximately 30–40% of the nodule’s volume. Additionally, multiple mildly enlarged lymph nodes were present in the mediastinum and left hilum, with the largest measuring 9 mm in short-axis diameter and demonstrating heterogeneous enhancement post-contrast. The combination of a spiculated margin despite well-defined borders and internal heterogeneity is highly suggestive of lung cancer (Figure 1A).

Gross pathological examination of the resected specimen identified a grayish-yellow, well-circumscribed nodule located 2.5 cm from the bronchial resection margin (Figure 1B). Histopathological examination revealed extensive histiocytic hyperplasia and deposition of lipoid-like material within the bronchial and alveolar lumina, as well as in the lung interstitium (Figures 1C–E). Immunohistochemical staining was positive for CK, TTF1, CD163, and CD34. Special staining was positive for PAS but negative for S100, HMB45, SMA, Ki67, PM, and GS (Figures 1F–I). The negative staining for S100 and HMB45 helps to rule out a granular cell tumor and melanoma, while the negativity for SMA argues against a myogenic neoplasm. The absence of staining for Ki67, PM, and GS further supports a non-neoplastic, inflammatory process, collectively consolidating the diagnosis of ELP.

A detailed postoperative history revealed that the patient had a long-standing history of allergic rhinitis. Crucially, it was only upon specific postoperative questioning that he reported excessive use of an over-the-counter nasal spray (Sato Nazal, containing naphazoline hydrochloride) for symptom relief. His usage pattern was significantly beyond the recommended dosage, amounting to 6 or more applications per day, with 3–5 sprays per use, over a period of several years. This history of chronic and excessive use of an oil-based spray was identified as the definitive etiology for the ensuing ELP. Unlike the classic histological presentation of scattered lipoid-laden macrophages, this case exhibited a localized nodular pattern, which, combined with the initially overlooked detailed medication history, contributed to the preoperative misdiagnosis. This case underscores the critical importance of meticulously correlating pathological findings with a comprehensive clinical history, including specific details of all over-the-counter medications, to achieve an accurate diagnosis and avoid unnecessary surgical procedures.

## Discussion

Lipoid pneumonia is a chronic inflammatory condition resulting from the abnormal accumulation of lipoids within the lungs, classified as either exogenous or endogenous. ELP can be further subdivided into chronic and acute forms. Chronic ELP is typically caused by the prolonged inhalation or aspiration of lipoid-based substances, such as mineral oil used for constipation or oily nasal drops employed for chronic rhinitis (1, 2). Our case, involving chronic and excessive use of an oil-based nasal spray, fits squarely within this etiology. Acute ELP, more frequently observed in children, often results from the accidental ingestion of hydrocarbons like kerosene or diesel (3).

The diagnosis of ELP is notoriously challenging due to its nonspecific clinical and radiological presentation, which often mimics lung cancer. Firstly, its symptoms are nonspecific and may include cough, sputum production, hemoptysis, chest pain, and fever. Some patients may even be asymptomatic, with the condition discovered incidentally on imaging (4). Secondly, radiological findings are equally nonspecific, often demonstrating ground-glass opacities, nodules, or consolidations that can mimic other pathologies, particularly lung cancer (5). This case exemplifies this challenge in its most deceptive form: a solitary pulmonary nodule. While ELP more commonly presents with diffuse opacities, the formation of a localized mass-like lesion, as seen here, can be attributed to a concentrated, chronic inflammatory response to the aspirated lipid. This leads to a robust fibrotic reaction and histiocytic aggregation that coalesces into a discrete nodule, effectively masquerading as a neoplasm on imaging. It is worth noting that such solitary pulmonary nodules can also be manifested in diseases such as granulomatous lung disease and pulmonary tuberculosis (6). The decisive factor leading to misdiagnosis in this case was not merely the suggestive radiology, but the critical omission of a detailed medication history during the initial clinical assessment. The patient’s excessive use of an oil-based nasal spray was only uncovered post-operatively. Without this pivotal etiological clue, the radiological findings were almost inevitably interpreted as malignancy, and the patient proceeded directly to resection. This oversight underscores that while advanced diagnostic techniques like transbronchial lung cryobiopsy (TBLC) (7) or intraoperative frozen section (8) can be valuable, their utility is precluded if the clinical suspicion for ELP is not first raised through meticulous history-taking. However, it is noteworthy that some studies suggest biopsy procedures may potentially exacerbate the condition, rarely leading to respiratory failure (9). Therefore, the risks and benefits of invasive diagnostic techniques must be carefully weighed. A multifaceted diagnostic approach integrating clinical history, imaging characteristics, and pathological findings is essential.

Currently, no standardized treatment guidelines exist for ELP. Management often involves conventional strategies such as supplemental oxygen therapy, bronchoalveolar lavage, glucocorticoids, and antibiotics, but treatment responses may be highly variable. Yasui et al. reported the successful management of severe, refractory ELP with systemic corticosteroids (10). In a pediatric case, repeated segmental bronchoalveolar lavage led to significant radiological improvement (11). Furthermore, the recruitment of intrapulmonary bronchopulmonary anastomoses has been shown to ameliorate hypoxemia in some ELP patients (12). These cases suggest the high heterogeneity of ELP treatment, so we still need to think long-term about its treatment.

Therefore, to prevent such misdiagnosis, we propose a more integrated diagnostic approach for solitary pulmonary nodules where clinical or radiological features are not entirely classic for malignancy. This method mainly involves a comprehensive clinical and radiological re-evaluation. Before invasive surgery, a systematic review of the patient’s medication history must be conducted, with a record and inquiry of all medications used, including over-the-counter nasal sprays, oils, and lubricants. The imaging features (for example, fat density on CT) should also be analyzed in detail. In addition, PET-CT should be used reasonably. Although FDG-PET-CT has been suggested to help distinguish, its utility may be limited (13, 14). The intense inflammatory response in ELP itself can cause significant FDG uptake, leading to false-positive results and possibly wrongly supporting a cancer diagnosis (15). Therefore, a positive PET finding must be interpreted very cautiously and in the complete clinical context.

This case delivers a critical lesson: ELP can present as a solitary nodule indistinguishable from lung cancer. The path to an accurate diagnosis begins not with advanced technology, but with a thorough and detailed clinical history. We strongly advocate for the integration of a systematic history-taking protocol into the evaluation of any suspicious pulmonary nodule. By doing so, clinicians can heighten their suspicion for ELP, utilize a stepwise diagnostic pathway to confirm it, and ultimately spare patients unnecessary and potentially harmful surgical procedures.

## Data Availability

The raw data supporting the conclusions of this article will be made available by the authors, without undue reservation.
