# Supplementary material for: Exogenous lipoid pneumonia masquerading as a pulmonary nodule: a case report
Source: Front Med (Lausanne). 2026 Jan 5;12:1723734. doi: 10.3389/fmed.2025.1723734 (PMC12812574; doi:10.3389/fmed.2025.1723734)
Supplement: Supplementary file 1 [file Table_1.docx]

**Materials and Methods**

**Immunohistochemical Analysis**
Formalin-fixed, paraffin-embedded (FFPE) tissue sections from the resected pulmonary nodule were used for immunohistochemical (IHC) staining. The staining was performed on an automated immunostainer (BenchMark XT, Ventana Medical Systems, USA) following the manufacturer's standard protocols. Briefly, the process involved dewaxing, rehydration, and antigen retrieval. The specific primary antibodies, their clones, dilutions, antigen retrieval methods, and manufacturers are summarized in Table 1. Appropriate positive and negative controls were run concurrently with each batch of staining. The positive controls included known positive tissue sections as per the antibody datasheet, while the negative controls were performed by omitting the primary antibody. Immunoreactivity was assessed by two experienced pathologists.

**Table 1. Antibodies and immunohistochemical staining details.**

| Antibody | Type | Dilution | Antigen Retrieval | Manufacturer |
| --- | --- | --- | --- | --- |
| CK | Monoclonal | 1:200 | Heat-induced, pH 9 | Dako, Denmark |
| TTF-1 | Monoclonal | 1:100 | Heat-induced, pH 9 | Roche, Switzerland |
| CD163 | Monoclonal | 1:400 | Enzyme-induced | Leica Biosystems, Germany |
| CD34 | Monoclonal | 1:100 | Heat-induced, pH 9 | Dako, Denmark |
| Ki-67 | Monoclonal | 1:200 | Heat-induced, pH 9 | Dako, Denmark |
| SMA | Monoclonal | 1:300 | Heat-induced, pH 9 | Dako, Denmark |
| S100 | Monoclonal | 1:2000 | Heat-induced, pH 6 | Dako, Denmark |
| HMB45 | Monoclonal | 1:100 | Heat-induced, pH 9 | Dako, Denmark |
